# Modified Stabilization Technique Following Resection of a Massive Cervical Infiltrative Lipoma with Spinal Compression in a Dog

**DOI:** 10.3390/ani16050747

**Published:** 2026-02-27

**Authors:** Hyung-Seok Seo, Hwi-Yool Kim, Jung-Moon Kim, Jun-Sik Cho, Sangyul Lee, Duhwan Park

**Affiliations:** Department of Veterinary Surgery, College of Veterinary Medicine, Konkuk University, Seoul 05029, Republic of Korea; gudtjrdldl@konkuk.ac.kr (H.-S.S.); kimjungm418@gmail.com (J.-M.K.); sangyul7918@gmail.com (S.L.); ppdh888@gmail.com (D.P.)

**Keywords:** infiltrative lipoma, cervical instability, ultra-high molecular weight polyethylene, stabilization

## Abstract

Infiltrative lipomas involving the upper cervical spine present a significant surgical challenge, as the extensive muscular resection required for tumor control often leads to severe structural instability. This report describes the surgical treatment of an infiltrative lipoma that extensively invaded the cervical area in a 9-year-old dog. Following wide surgical debulking, a dual-plane stabilization strategy was employed. Ventral stabilization was attempted using standard C1–C2 transarticular screw fixation, while a modified dorsal stabilization technique anchored the occipital protuberance to the C2 spinous process using an ultra-high molecular weight polyethylene (UHMWPE) suture construct, combined with nuchal ligament reconstruction. Follow-up at 78 days revealed failure of the ventral implants, characterized by immediate improper positioning of the right screw and subsequent migration of the left screw. Despite these complications and confirmed tumor recurrence, the patient maintained normal neurological function and clinical cervical stability. This clinical course was suggestive of fibrous or early osseous union at the atlantoaxial joint. These findings suggest that the modified dorsal stabilization technique provided critical biomechanical support, effectively compensating for the compromised ventral fixation, and may represent a potential surgical option for managing extensive occipito-cervical instability in dogs.

## Simple Summary

This report describes a dog with a large infiltrative lipoma involving the upper cervical spine, requiring an extensive tumor resection that resulted in marked upper cervical instability. Dual-plane stabilization was performed using ventral transarticular screws and a braided ultra-high molecular weight polyethylene (UHMWPE) suture-based dorsal stabilization. Despite ventral implant failure and tumor recurrence, the dog maintained normal neurological function and cervical stability. These findings indicate that the dorsal technique may be a useful option for managing severe occipito-cervical instability in dogs.

## 1. Introduction

Infiltrative lipoma is a histologically benign tumor characterized by local invasion into surrounding tissues such as muscle and connective tissue [1,2,3]. Infiltrative lipoma predominantly affects the extremities [4,5,6]. However, in some cases, they invade the axial skeleton, including the bone and the vertebral canal [7,8,9,10,11,12]. When infiltrative lipoma involves the vertebral spine, it may cause structural compromise and neurological deficits.

The standard treatment for infiltrative lipoma is wide surgical excision to minimize local recurrence. However, the infiltrative nature of the tumor often makes defining clear surgical margins difficult [2,3,8]. In cases where complete excision is challenging, radiation therapy has been reported as an effective adjunctive therapeutic option to control tumor regrowth [13,14]. Nevertheless, when the primary goal is maximizing tumor resection, aggressive resection of the surrounding soft tissues is often necessitated. This challenge is particularly pronounced when the tumor involves the axial skeleton. In these regions, the extensive resection of essential supporting musculature and ligaments required for tumor control can severely compromise structural integrity. Since the spinal stabilizing system relies heavily on the active subsystem provided by paraspinal muscles and the passive subsystem provided by ligaments, the loss of these structures inevitably predisposes the affected region to severe instability [15,16].

However, established veterinary stabilization techniques to address such extensive cervical instability are currently limited. Therefore, successful management depends not only on effective tumor debulking but also on devising a robust strategy for restoring biomechanical stability.

This case report describes the surgical management of a massive cervical infiltrative lipoma, detailing a dual-approach strategy for tumor debulking and the application of a modified occipito-cervical stabilization technique to address the resulting instability.

## 2. Case Description

### 2.1. Signalment, History, and Clinical Findings

A 9-year-old, 9.7 kg intact female mongrel dog was presented for evaluation of difficulty lifting its head. The dog had been adopted 5 years prior, at which time a cervical mass was already noted. The mass gradually enlarged by approximately 20% over the period, according to the owner’s observation, and the dog developed a persistently lowered head posture.

Physical examination revealed a firm, non-movable mass measuring approximately 12.5 × 11 × 11.3 cm, extending from the right dorsal to ventral aspects of the neck. Pain was elicited during ventral flexion, with reduced dorsal extension and right lateral bending. Neurological examination revealed mildly decreased proprioceptive positioning in the forelimbs; however, no obvious gait abnormality was observed, and spinal reflexes were normal.

### 2.2. Diagnostic Evaluation

CBC and serum biochemistry results were within normal limits. Fine-needle aspiration revealed low cellularity with scattered adipocytes, suggesting the possible lipoma.

**Figure 1:**
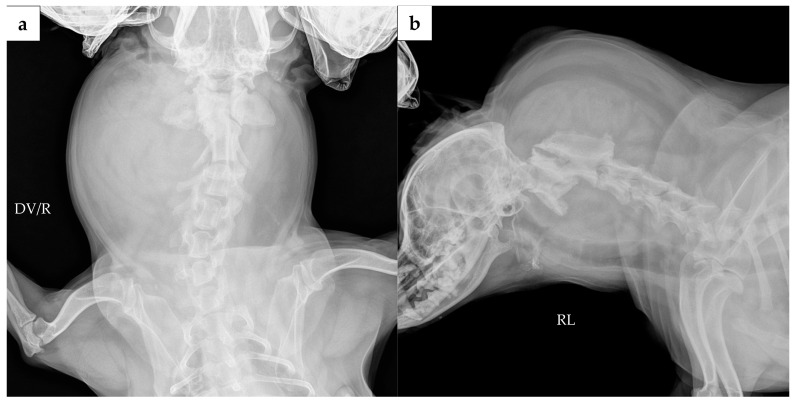
Preoperative radiographic images: (**a**) Dorsoventral (DV) view shows a large soft tissue density in the right cervical region, causing mild rightward malalignment of the cervical vertebral column; (**b**) right lateral (RL) view shows ventral deviation of the trachea and the presence of the soft tissue mass. Abbreviations: DV, dorsoventral; R, right; RL, right lateral.

**Figure 2:**
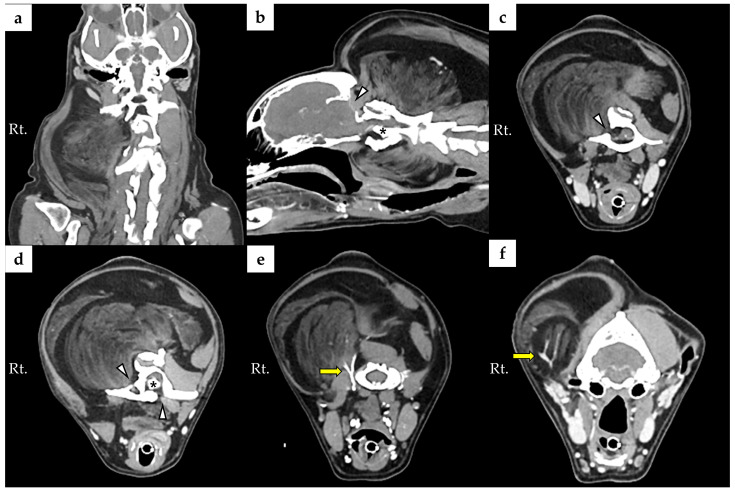
Preoperative computed tomography showing poorly encapsulated mass with ill-defined margins, mixed with surrounding muscle: (**a**) Coronal view showing that the surrounding musculature was significantly compromised by the tumor; (**b**) sagittal view showing the ventral extension of the tumor and displacement of the dens (asterisk), together with bone lysis of the occipital bone (arrowhead); (**c**) transverse CT image at the level of the atlas showing that the lipoma occupies approximately 80% of the cross-sectional soft tissue area and extends into the vertebral canal through the right lamina of C2 (arrow head), resulting in spinal cord compression; (**d**) transverse view shows the bone lysis on the right wing and ventral pedicle of the atlas (arrowheads) and dorsal displacement of the dens (asterisk); (**e**) feeding artery (yellow arrow) arising from the internal carotid artery; (**f**) feeding artery (yellow arrow) arising from the external carotid artery. Abbreviations: Rt, right.

Cervical radiographs revealed a 125 × 110 × 113 mm massive soft tissue density (Figure 1). The cervical vertebral column showed mild rightward malalignment on the dorsoventral projection, and the trachea was displaced ventrally on the right lateral projection. A computed tomography (CT) scan was performed using a CT scanner (Aquilion Lightning; Canon Medical Systems, Otawara, Japan). CT scanidentified a poorly encapsulated mass intermingled with adjacent muscle, occupying approximately 80% of the soft tissue at the C1 level (Figure 2a). The mass extended ventrally between the C1–C2 articular facets, displacing the dens dorsally (Figure 2b,d). Based on this displacement, the ventral compression index (VCI) was 0.8 (diagnostic cutoff: ≥0.16 in the extended head position), supporting a diagnosis of atlantoaxial instability.

Consistent with this instability, bony proliferation was noted on the ventral aspect of C1 and on the C2 spinous process. Bone lysis was also present in the occipital bone and in the right lamina, right wing, and ventral pedicle of C1 (Figure 2b–d, arrowheads). The extension of the mass through the right lamina of C2 was also associated with spinal cord compression (Figure 2c).

**Figure 3:**
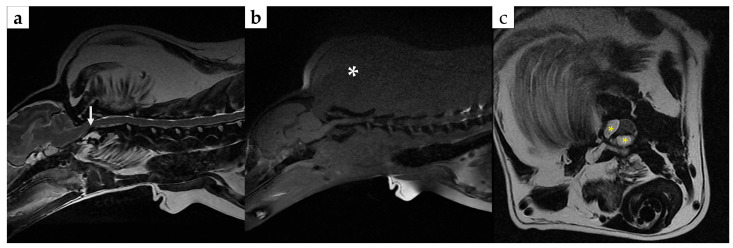
Magnetic resonance imaging of the cervical region: (**a**) Sagittal T2-weighted MRI image showing a ventral signal void (arrow), considered to reflect narrowing/compression and accelerated CSF flow at the C1–C2 level; (**b**) sagittal T1-weighted fat-suppressed MRI image showing marked signal suppression of the mass (asterisk) compared with the corresponding non–fat-suppressed images, supporting a fat-containing (lipomatous) lesion; (**c**) transverse T1-weighted MRI image at the C1–C2 level showing right-sided and ventral extension of the mass into the vertebral canal (yellow asterisk).

Magnetic resonance imaging (MRI) was performed using a 1.5-Tesla system (Signa Hdxt, GE Medical Systems, Milwaukee, WI, USA). On T2-weighted imaging, ventral flow-void phenomena at the level of C1 and C2 were observed; this finding was considered to reflect ventral subarachnoid space narrowing/compression secondary to dorsal displacement of the dens (Figure 3a). Fat-suppressed sequences were also obtained (T1-weighted fat suppression and T2-weighted fat suppression). On the fat-suppressed T1-weighted images, the mass demonstrated marked signal suppression compared with the corresponding non–fat-suppressed T1-weighted images, which was considered supportive of a fat-containing lesion. On the fat-suppressed T2-weighted images, no findings suggestive of intramedullary edema or inflammation were identified. Right-sided and ventral extension into the vertebral canal of the mass was also observed between the level of C1 and C2 (Figure 3c). The degree of maximal spinal cord compression was approximately 17%. Because contrast-enhanced MRI was not performed, enhancement characteristics could not be evaluated.

### 2.3. Assessment and Surgical Plan

Based on these findings, a presumptive diagnosis of cervical infiltrative lipoma with extension to the vertebral canal and associated spinal cord compression was made.

Surgical debulking and stabilization were planned. Due to the extensive infiltration of the mass, wide resection of the cervical muscles was deemed necessary. This extensive resection was expected to cause severe iatrogenic occipito-cervical instability. Therefore, a dual-plane stabilization strategy was devised. The plan consisted of ventral stabilization using a standard C1–C2 transarticular screw technique and a modified dorsal stabilization technique connecting the occipital protuberance to the C2 spinous process using braided ultra-high molecular weight polyethylene (UHMWPE) suture (Ligafiba, Veterinary Instrument, Sheffield, UK).

### 2.4. Anesthesia and Surgical Procedure

Preanesthetic medication included intravenous midazolam (0.2 mg/kg, Bukwang midazolam inj., Bukwang Pharm, Seoul, Republic of Korea) and a fentanyl bolus (2 mcg/kg, Hana Fentanyl Citrate inj., Hana Pharm, Seoul, Republic of Korea) administered as pre-induction analgesia (loading dose) to provide rapid-onset pain control. General anesthesia was induced with propofol (3 mg/kg, Anepol inj., Hana Pharm, Seoul, Republic of Korea) and maintained with isoflurane (Ifran Liq. Hana Pharm, Seoul, Republic of Korea), while intraoperative analgesia was provided via a fentanyl constant rate infusion (CRI).

The surgical management consisted of a three-stage procedure necessitating two intraoperative repositioning maneuvers to facilitate circumferential access to the cervical spine. The sequence proceeded as follows: initial ventral debulking (dorsal recumbency), followed by dorsal debulking and stabilization (ventral recumbency), and concluding with ventral stabilization (dorsal recumbency).

#### 2.4.1. Ventral Debulking

**Figure 4:**
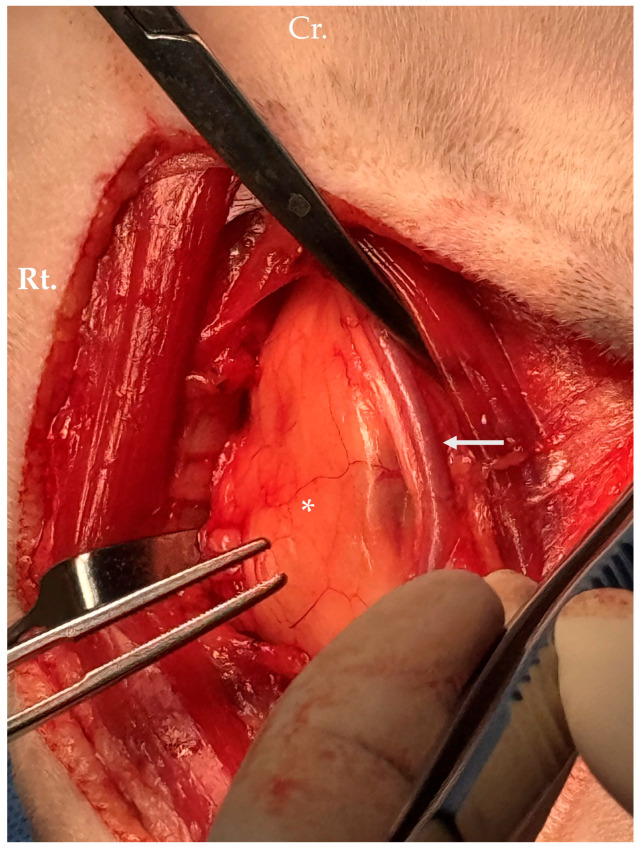
Intraoperative photograph of mass debulking via ventral midline approach. The lipoma (asterisk) was adherent to the left carotid sheath (arrow), highlighting the close anatomical relationship between the mass and major cervical vascular structures. Abbreviations: Rt., right.; Cr., cranial.

Through a ventral midline approach, tumor debulking was performed while preserving major structures, including the carotid sheath and recurrent laryngeal nerve (Figure 4). The longus colli and longus capitis muscles were severely infiltrated and were resected to the greatest extent possible.

#### 2.4.2. Dorsal Debulking and Stabilization

**Figure 5:**
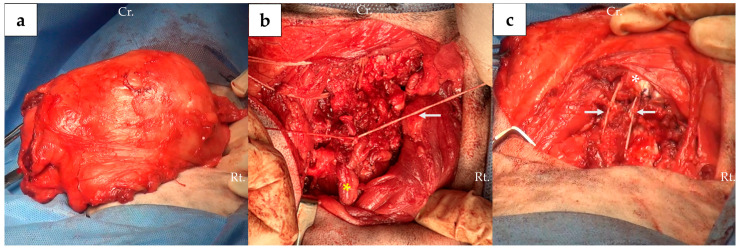
Intraoperative images: (**a**) Cervical mass was exposed via a dorsal approach; (**b**) reattachment of the transected nuchal ligament (yellow asterisk) to the spinous process of C2 using Ligafiba (white arrow); (**c**) Ligafiba (white arrow) was passed through the occipital protuberance (white asterisk) and spinous process of C2. Abbreviations: Rt., right.; Cr., cranial.

The patient was repositioned into ventral recumbency for a dorsal approach. Upon dorsal approach, most of the key stabilizing structures, including the Rectus capitis major, Semispinalis capitis, Longissimus capitis muscles, and the Nuchal ligament, specifically near its insertion on the C2 spinous process, were found to be infiltrated by the tumor. These infiltrated portions were resected as much as possible during debulking (Figure 5a).

**Figure 6:**
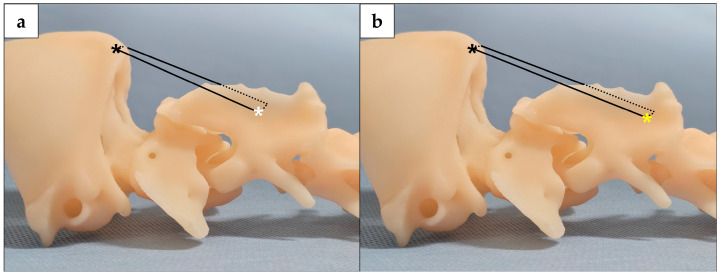
A 3D-printed bone model, created from this case’s preoperative CT data, was used to confirm the locations of bone proliferation and lysis, which directly informed the choice of surgical technique and fixation sites. Ligafiba sutures were utilized to secure the OPh (black asterisk) to SPh1 ((**a**), white asterisk) and SPh2 ((**b**), yellow asterisk), respectively.

Prior to stabilization, the bony proliferation on the C2 spinous process was removed using a rongeur and a burr. Stabilization was then performed by drilling one hole (OPh) in the occipital protuberance and two holes (SPh1, SPh2) in the C2 spinous process using a 1.2 mm Kirschner wire (Jeil Medical Co., Ltd., Seoul, Republic of Korea). To prevent intracranial damage, the hole in the occiput was planned at its thickest point, as predetermined by CT measurements. The occipital hole (OPh) was created approximately 5 mm cranial to the caudal edge of the occipital protuberance. The second hole in the C2 spinous process (SPh2) was created with reference to the nuchal ligament insertion, approximately 8 mm ventral to the cranial edge of the insertion site, and SPh1 was created approximately 5 mm cranial to SPh2 (Figure 6).

Stabilization was achieved by passing a braided UHMWPE suture (Ligafiba) between OPh and SPh1, and between OPh and SPh2 (Figure 5c and Figure 6a,b). Fixation was completed using a hand-tied technique; for each strand, one surgeon’s knot followed by one square knot was applied. Because excessive extension at the time of fixation may impose undue tension on the sutures and increase the risk of premature suture failure, the head and neck were maintained in a neutral position during tensioning. Under these conditions, tension was adjusted so that the two Ligafiba strands (OPh–SPh1 and OPh–SPh2) were tightened as evenly as possible.

The nuchal ligament was reattached to SPh2 using a Krackow suture technique, also utilizing Ligafiba (Figure 5c). Fixation of this construct was completed using the same hand-tied technique, with one surgeon’s knot followed by one square knot, while the head and neck were maintained in a neutral position. The dorsal surgical site was closed in layers.

#### 2.4.3. Ventral Stabilization

The patient was then repositioned back into dorsal recumbency. During repositioning, the dorsal occipito-cervical construct was thought to help maintain craniocervical alignment and may have reduced the risk of excessive cervical flexion. Re-entering the initial ventral approach, the C1–C2 articular cartilage was removed using a burr to promote postoperative arthrodesis. The bony proliferation on the ventral side of C1 was also removed at this time. No cancellous bone graft was applied in this case to minimize additional surgical time and donor-site morbidity, given the prolonged operative time and limited residual soft-tissue coverage after extensive debulking. Additionally, a nerve hook was used to remove the ventrally invasive tumor located between the articular surfaces. Atlantoaxial reduction was required prior to ventral transarticular screw placement. Two standard C1–C2 transarticular screws (1.5-mm diameter, 14-mm long cortical screws; Arix, Jeil Medical Co., Ltd., Seoul, Republic of Korea) were placed in lag fashion. A Penrose drain (Sewoon Medical Co., Ltd., Seoul, Republic of Korea) was placed, and the surgical site was closed in layers.

### 2.5. Postoperative Course and Follow-Up

**Figure 7:**
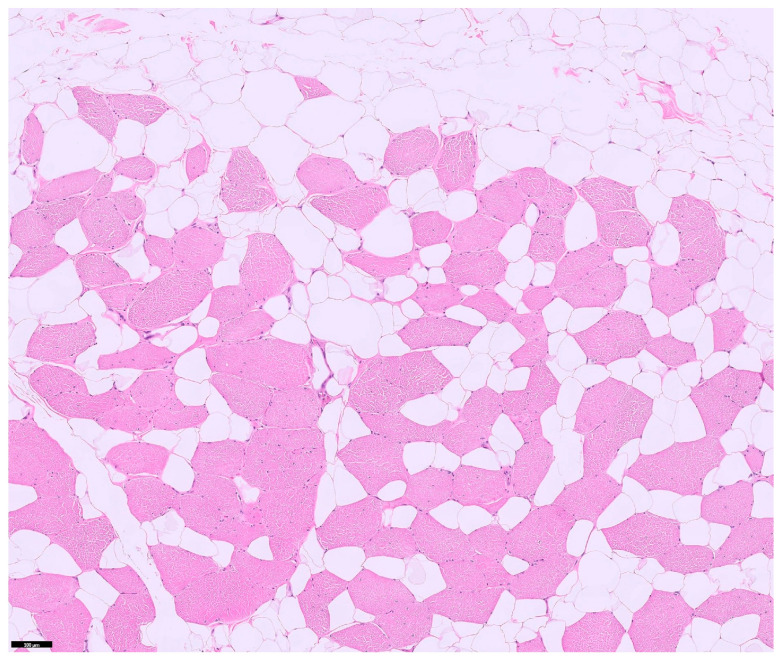
Histopathological appearance of the neck mass (HE stain ×100). Well-differentiated adipose tissue, which is exhibiting multifocal haphazard replacement and infiltration of regional striated muscle bundles.

Histopathological analysis of the excised tissue confirmed the diagnosis of infiltrative lipoma, characterized by well-differentiated adipocytes infiltrating skeletal muscle bundles (Figure 7).

**Figure 8:**
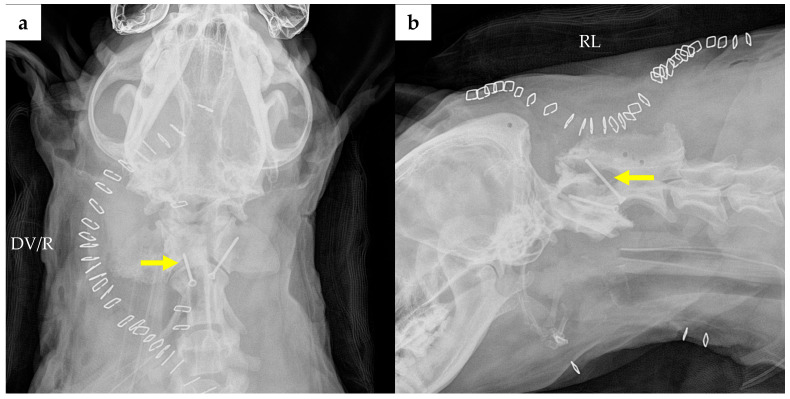
Postoperative radiographic images on operation day: (**a**) Dorsoventral (DV) view shows suspected malpositioning of the right transarticular screw (yellow arrow); (**b**) right lateral (RL) view shows that the right transarticular screw (yellow arrow) failed to engage the atlas. The trachea, which had been ventrally displaced by the mass preoperatively, appears to have improved alignment following surgery. Abbreviations: DV, dorsoventral; R, right; RL, right lateral.

Immediate postoperative radiography revealed improper positioning of the right transarticular screw, which failed to engage the atlas (Figure 8). Reoperation was considered; however, given the prolonged initial procedure (approximately 8 h of surgical time and 11 h of anesthesia) and the delayed recovery from anesthesia, immediate re-anesthetization was considered to carry a higher risk. In particular, despite vasopressor support, mean arterial pressure remained below 60 mmHg for a prolonged period during anesthesia, raising concerns regarding hemodynamic stability with repeat anesthesia. At that time, no progressive neurological deficits were observed, and a conservative observation approach with close clinical monitoring and follow-up imaging was elected. These considerations were discussed with the owner, and the owner declined reoperation.

The patient was discharged on postoperative day 13. The owner was instructed to apply a neck brace for 2 weeks post-discharge and to strictly restrict the dog’s activity (cage rest with leash walks only) for at least 4 weeks. No neurological deficits were observed at the time of discharge.

**Figure 9:**
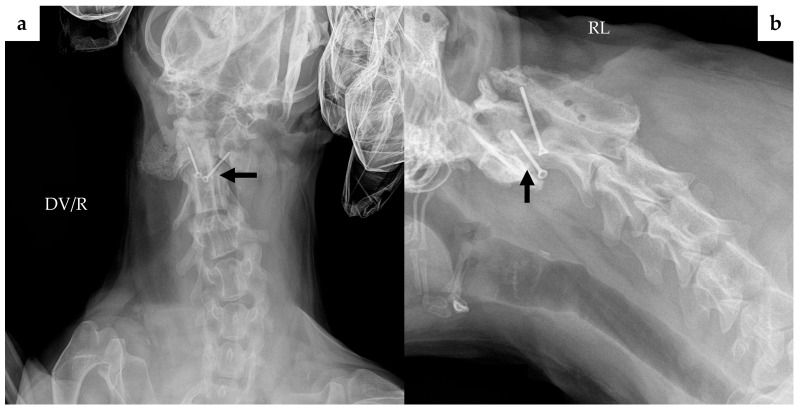
Follow-up radiographic images on POD 78. (**a**) Dorsoentral (DV) view and (**b**) right lateral (RL) view. Migration of the left transarticular screw (arrow) is evident in both views. Abbreviations: DV, dorsoventral; R, right; RL, right lateral.

**Figure 10:**
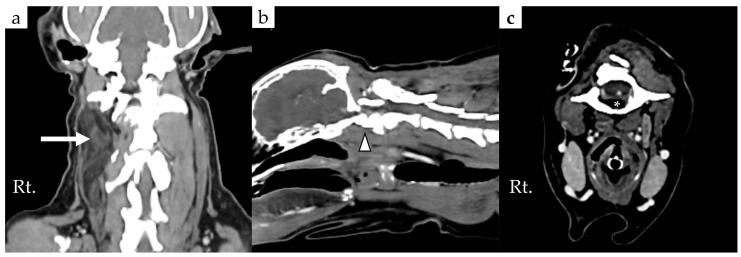
Follow-up computed tomography (CT) images on postoperative day (POD) 78: (**a**) Coronal view of the cervical region showing a soft tissue mass (arrow) along the right side, compatible with residual or recurrent lesion; (**b**) sagittal view shows an area where partial osseous continuity may be present at the atlantoaxial joint (arrowhead), which may indicate early or partial bone union between C1 and C2; (**c**) transverse view at the level of C1 shows that the extent of the soft tissue mass on the right side of vertebral canal appears reduced compared to preoperative imaging, while ventral extension of the mass (asterisk) remains evident. Abbreviations: Rt, right.

At the POD 78 recheck, the neurological examination remained normal. Cervical range of motion was unremarkable on extension, while mild stiffness was noted on flexion; however, no pain was elicited during movement or palpation, and overall cervical stability was maintained. No external mass was visible, though firmness was palpated at the surgical site. Follow-up radiography revealed migration of the initially well-positioned left transarticular screw (Figure 9). Despite this implant complication, follow-up CT demonstrated an area where partial osseous continuity may be present between C1 and C2, suggesting possible partial bony bridging at the atlantoaxial joint (Figure 10b). Regarding the tumor status, residual or recurrent tissue was confirmed in the right cervical area (Figure 10a). Although the ventral invasion of the tumor remained present, it demonstrated slight regression. In contrast, the extent of the soft tissue mass on the right lamina of C1 appeared reduced compared to previous imaging (Figure 10c).

## 3. Discussion

This case describes a rare presentation of a massive infiltrative lipoma involving the occipital bone and the upper cervical spine. In rare cases, infiltrative lipomas can invade bone and neural structures. Extension into the vertebral canal, causing spinal cord compression, has been sporadically reported in the veterinary literature, but these cases typically involve the thoracolumbar or mid-cervical spine [7,8,9,10,11,12]. To the authors’ knowledge, such extensive infiltration involving the occipital region and the atlas and axis has not been previously described. This specific anatomical involvement presents a unique surgical challenge. The lack of encapsulation and the extensive infiltration of tumor cells into essential spinal musculature make defining clear surgical margins difficult. Consequently, high local recurrence rates ranging from 36% to 50% have been reported even with aggressive surgical resection [2,3]. Consistent with these data, residual and/or recurrent tumor tissue was suspected in the present case at the 78-day follow-up after extensive surgery.

The spinal stabilizing system is conceptually divided into three subsystems: the passive musculoskeletal subsystem, the active musculoskeletal subsystem, and the neural control subsystem [16]. In the present case, stabilizing subsystems were severely compromised due to the extensive resection of the cervical musculature and ligament required for tumor debulking. Despite the extensive infiltration of the tumor, it may also have provided a degree of mechanical support to the cervical column. The standard ventral stabilization technique using transarticular screws requires removal of the articular cartilage to promote arthrodesis, which may transiently compromise passive stability in the immediate postoperative period until a fibrous or osseous union develops [17,18,19]. If ventral stabilization had been initiated during the initial ventral approach, preparation of the C1–C2 articular surfaces for arthrodesis (including articular cartilage removal) could have transiently reduced passive stability at the atlantoaxial joint prior to securing dorsal stabilization, thereby increasing the risk of instability during subsequent repositioning. Because dynamic muscular support was already expected to be reduced due to extensive resection and general anesthesia, maintaining passive stability during repositioning was considered particularly important. We determined that repositioning the patient in such a highly unstable state would pose a substantial risk of iatrogenic fracture or atlantoaxial luxation. Therefore, the application of the transarticular screws was intentionally deferred until the final stage of the surgery.

In quadrupeds, the spine functions biomechanically as a suspension mechanism rather than a vertical column of bipeds, creating a unique loading pattern where gravity exerts significant ventral forces [20,21,22]. The integrity of the dorsal column is critical for maintaining stability against these gravitational forces [20]. In this context, the nuchal ligament acts as a passive tension band, supporting the head’s weight and conserving muscular energy [15,23,24,25]. In this case, the extensive resection of the dorsal extensor muscles and the nuchal ligament was expected to result in functional insufficiency, potentially leading to an inability to elevate the head and increasing the risk of subsequent neurological sequelae [26]. Therefore, dorsal stabilization was deemed essential to compensate for this muscular and ligamentous deficit. To address this, we employed a modified occipito-cervical fixation technique utilizing the external occipital protuberance (EOP) and the C2 spinous process as the primary fixation points. The EOP was selected as a cranial anchor because it is recognized in human literature as the thickest and strongest portion of the occiput, making it ideal for screw or suture placement [27,28,29]. The C2 spinous process was selected as the caudal anchor because it provides sufficient bone stock for adequate purchase. By spanning the occiput and C2 with a dorsal tension-band construct, this configuration was intended to provide cranially directed traction to counteract ventral head-drop tendencies and to promote a mild ventral tilt of the axis, which may help reduce the risk of dorsal dens displacement. Furthermore, by reattaching the remaining nuchal ligament caudally to the C2 spinous process, we aimed to restore cervical stability through a continuous dorsal tension-band system centered on the axis.

These fixation points were coupled using Ligafiba, a braided suture material composed of ultra-high molecular weight polyethylene (UHMWPE). A clinically relevant precedent for this concept has been reported in a cat with atlanto-occipital instability, where dorsal stabilization using divergent tension bands made of a synthetic UHMWPE-based ligament prosthesis (OrthoFiber) anchored between the occipital nuchal crests and the C2 spinous process [30]. Compared to traditional orthopedic wire, Ligafiba offers superior flexibility and ease of handling [31]. Furthermore, it demonstrated the highest ultimate tensile strength and greatest resistance to cyclic elongation among evaluated materials, suggesting the potential to provide immediate multidirectional stability [32]. In the present case, the dorsal construct required controlled and symmetric tensioning through drilled osseous holes while minimizing implant bulk in a region with limited soft-tissue coverage after extensive debulking. In addition to its reported mechanical performance, Ligafiba can be considered advantageous over traditional wire because it is easier to handle and allows more controlled tensioning and knot tying, and it may reduce the risk of wire cut-through (“cheese-wiring”) in small or thin osseous structures. Nevertheless, long-term verification is required to establish durability and complication rates.

Potential complications associated with the nonabsorbable braided suture constructs should also be considered. In human orthopedic surgery, delayed mass-like foreign-body reactions related to UHMWPE-based sutures and local bony reactions (including granuloma formation and osteolysis) through bone tunnels have been reported, suggesting a potential risk in techniques that rely on drilled osseous holes [33,34]. Moreover, UHMWPE-based braided sutures may be more susceptible to bacterial adherence (and subsequent colonization) than nylon [35]. In the present case, no clinically apparent Ligafiba-related local complications were identified during the available follow-up period.

Clinical signs of atlantoaxial instability (AAI) most commonly involve neck pain, with neurological deficits ranging from mild postural abnormalities to tetraplegia, depending on the severity of spinal cord damage [17,36,37]. In the present case, the patient exhibited severe pain upon ventral flexion and decreased proprioceptive reactions in the forelimbs. On CT images, VCI was calculated as the ratio of the ventral atlanto-dental interval to the dorsal atlanto-dental interval, measured along a line drawn between the midpoints of the atlas ventral and dorsal arches. The VCI was 0.8, which markedly exceeds the proposed diagnostic cutoff values for AAI (≥0.16 in extension and ≥0.20 in flexion), providing additional objective support for atlantoaxial instability in this dog [38]. On T2-weighted spin-echo imaging, a cerebrospinal fluid (CSF) signal-void (flow-void) sign may be observed when CSF flow is rapid or turbulent; however, it can also be seen in dogs with various conditions and even without obvious morphologic abnormalities [39]. Accordingly, in the present case, the ventral CSF signal void at the C1–C2 level was interpreted cautiously as an indirect finding that may reflect increased CSF velocity through a narrowed ventral subarachnoid space, in conjunction with the anatomic evidence of ventral subarachnoid space narrowing secondary to dorsal displacement of the dens. Reported spinal infiltrative lipomas show a wide spectrum of clinical severity [7,10,11,12]. The pattern observed in this dog appears to fall toward the milder end of that spectrum and does not represent a clearly typical presentation. Neurological abnormalities in this dog were non-lateralizing, with decreased proprioceptive reactions in both forelimbs and no clear right-sided predominance despite the right-sided extension of the mass on imaging. Previous work has shown that the degree of clinical dysfunction cannot be predicted solely from the percentage of spinal cord compression [40]. The chronic and slowly progressive nature of the infiltrative lipoma in this dog may, at least in part, explain why the clinical signs were dominated by cervical pain and postural abnormalities rather than acute and severe neurological deficits [41].

Surgical intervention is generally indicated for affected dogs to align and stabilize the joint, thereby preventing further spinal cord damage and catastrophic recurrence. Both ventral and dorsal stabilization techniques have been described for the surgical management of AAI in dogs [17,19,36,37,42,43,44,45]. Dorsal stabilization techniques include atlantoaxial wiring, the nuchal ligament technique, and the tension-band construct. However, these methods commonly rely on the dorsal arch of the atlas for fixation, and preoperative CT in this case demonstrated bone lysis involving the atlas, raising concern for limited anchor strength and a potentially increased risk of iatrogenic atlas fracture. Therefore, a commonly described dorsal AAI technique was not selected as the primary C1–C2 stabilization method in this patient. Standard ventral stabilization techniques include transarticular pins or screws, methods combining multiple implants with polymethylmethacrylate (PMMA), and ventral plate fixation [17,19,37,44,45,46]. However, these procedures are technically challenging and associated with high complication rates, reported to be as high as 38% to 53% [17,46]. In this patient, the application of multiple implants or PMMA was deemed excessively risky. Preoperative imaging revealed bone lysis in the left wing and right lamina of the atlas and the pedicle of the axis, suggesting that the bone was weakened by the tumor. Placing multiple implants in such compromised bone carried a high risk of iatrogenic fracture. Pedicle screw augmentation (e.g., C1 and/or C2) was also considered; however, given the CT evidence of bone lysis involving the atlas and axis and the limited implant purchase expected in the distorted anatomy, additional screw placement was judged to increase the risk of iatrogenic fracture and implant-related complications in this patient. Furthermore, due to the minimal soft tissue remaining after extensive debulking, the exothermic reaction and bulk of PMMA posed a significant risk of inducing fibrous adherence or thermal injury to vital adjacent structures, such as the esophagus and trachea [47,48]. Ventral plate fixation was also considered impractical in this case because severe ventral bony proliferation and distortion were expected to prevent adequate plate contouring and seating. The mass extended ventrally into the vertebral canal, necessitating a ventral approach to address the ventrally invasive component and to remove tissue located between the C1–C2 articular surfaces. The articular surfaces were prepared (including cartilage removal) to facilitate postoperative arthrodesis; therefore, ventral stabilization was selected to support the joint during the healing period. Consequently, the transarticular screw technique was selected as the sole ventral stabilization method. However, transarticular screws placed in lag fashion are subject to failure, especially during implantation, because the small volume of bone in the axis available for screw engagement permits no margin for error [17,18,42]. This inherent difficulty was compounded by the chronic anatomical distortion in our case. As anticipated with these risks, both ventral implants eventually failed: the right screw was improperly positioned immediately postoperatively, and the left screw migrated by postoperative day 78. Implant migration typically occurs more than 3 weeks postoperatively; if sufficient fibrous or osseous union develops during this period, additional surgical intervention may not be necessary [17]. In this case, despite the documented failure of both ventral implants, the patient maintained cervical stability and demonstrated continued neurological improvement. Furthermore, follow-up imaging revealed signs consistent with partial union. This outcome is consistent with the dorsal occipito-cervical stabilization having provided important support, allowing adequate time for union to form at the C1–C2 articular surfaces before the ventral implant complications could lead to clinical instability.

This case report has several limitations. First, long-term follow-up beyond postoperative day 78 was not available; therefore, the risks of delayed neurological deterioration or further tumor progression remain unknown. Second, the absence of immediate postoperative CT imaging precludes precise assessment of the extent of resection, and it cannot be determined whether the tissue identified on day 78 represented residual tumor or early local regrowth. Third, the mechanical effect of the dorsal stabilization construct cannot be evaluated in isolation. External support from the cervical brace, together with potential passive stabilization provided by residual or recurrent soft tissue, may have contributed to maintenance of craniocervical alignment, and the relative contribution of each factor cannot be objectively quantified. The observed short-term clinical stability is therefore best interpreted as the net result of the dorsal construct, any temporary support from surrounding tissues, and early healing responses. Although the ventral fixation failed because of initial malpositioning and subsequent screw migration, no new neurological deficits were observed during the follow-up period. While causality cannot be established in a single case, this clinical course is at least consistent with the modified dorsal occipito-cervical stabilization, providing a potential biomechanical contribution to maintaining functional alignment at the craniocervical junction.

## 4. Conclusions

Complete surgical excision of infiltrative lipomas involving the cervical spine is challenging and often necessitates extensive muscular resection, which can result in severe iatrogenic instability. In this case, despite the failure of ventral stabilization, clinical cervical stability was effectively maintained during the short-term follow-up. This outcome suggests that the modified dorsal stabilization technique—anchoring the occipital protuberance to the C2 spinous process and reconstructing the nuchal ligament—may have provided supplementary biomechanical support. Therefore, this technique may be a feasible adjunctive stabilization method for managing extensive occipito-cervical instability in dogs, particularly in cases where ventral fixation is limited or compromised; however, its long-term durability and complication profile require further investigation.

## Data Availability

The data presented in this study are available in the article.
